# Supplementary material for: Cystic Fibrosis Newborn Screening: A Systematic Review-Driven Consensus Guideline from the United States Cystic Fibrosis Foundation
Source: Int J Neonatal Screen. 2025 Apr 2;11(2):24. doi: 10.3390/ijns11020024 (PMC12015897; doi:10.3390/ijns11020024)
Supplement: Supplementary file 1 [file IJNS-11-00024-s001.zip › IJNS-3488954-supplementary.pdf]

Supplemental Methods

Methods and Data Availability Statement

Cystic Fibrosis Foundation Guideline Committee

The Cystic Fibrosis Foundation (CFF) invited committee members to join the guideline committee in 2022, and those that accepted went through a conflict-of-interest review. The multidisciplinary committee had representation from across the US and was made up of five cystic fibrosis (CF) clinicians and researchers, three parents of children with CF who had newborn screening (NBS), three state NBS program experts, a primary care pediatrician, a genetic counselor, and CF Foundation staff. The committee collaborated to develop six systematic reviews on issues including immunoreactive trypsinogen, cystic fibrosis transmembrane conductance regulator (*CFTR*) molecular testing, and results notification practices. Each systematic review was approved by the committee, and protocols were registered in the International Prospective Register of Systematic Reviews (PROSPERO) prior to the commencement of the literature review.[1,2,3,4,5,6]

PICO strategy, application and timeline

Population, Intervention, Comparison, and Outcome (PICO) questions were developed and agreed upon by the full committee during virtual meetings to identify the NBS processes that impact equity, sensitivity, and timeliness of diagnosing infants with CF. Cost-effectiveness and resource allocation were not addressed. The identification of infants with *CFTR*-related metabolic syndrome (CRMS) or CF Screen Positive, Indeterminate Diagnosis (CFSPID) was not addressed as there are prior CFF guidelines for these conditions.[7] Six systematic reviews were conducted between November of 2023 and May of 2024. Two reviewers were assigned to each PICO question and reviewed the literature at the title, abstract, and full text level. The articles were reviewed using the Rayyan platform and spreadsheets were used to extract data at the full text level to be used as supporting evidence. To receive copies of the data extraction tool used during the full text review for each PICO question, contact the corresponding author. Reviewers met to re-review excluded articles and adjudicate disagreements. The parent representatives on the committee were recruited through CF Foundation Community Voice, a program for people with CF (PwCF) and their family members to shape CF research and care by sharing their lived experiences and perspectives. Two of the three parent representatives on the committee agreed to participate in the literature review and were paired with clinicians who had experience conducting systematic literature reviews. A medical research librarian (QEW) collaborated in developing the literature search and uploaded the resulting abstracts on the Rayyan platform (see “search strategies” section for further details). After the abstract screening, the librarian provided all articles that were designated “include” or “maybe” for full text review. Committee members also added hand selected articles that were not returned in the systematic review but that had impact on the PICO question. A standardized data extraction tool was used to review the resulting articles and recommend whether to include or not include the articles. Disagreements were adjudicated by reviewers. Each PICO review team created recommendation statements based on the included

literature. The committee convened to vote on recommendation statements in July 2024. An 80% consensus was the *a priori* threshold for approval, and all statements received 100% approval from the committee.

Search Strategies

For each of the six PICO questions, the guideline committee collaborated with a medical research librarian (QEW) to create detailed search strategies addressing specific aspects of CF screening in newborns. For PICO 1, the search focused on CF, newborns, immunoreactive trypsinogen, and false negatives. PICO 2 targeted CF, newborns, immunoreactive trypsinogen, and CFTR sequencing. PICO 3 emphasized CF, newborns, immunoreactive trypsinogen, cutoff values, and screening algorithm sensitivity. In PICO 4, the focus was on CF, newborns, immunoreactive trypsinogen, and DNA variant panels, while PICO 5 explored CF, newborns, immunoreactive trypsinogen, and CFTR DNA testing. PICO 6 centered on CF, newborns, screening, notification, and results. Searches were performed in Medline (Ovid), Cochrane Library (Wiley), Embase (Elsevier), Web of Science (Thomson Reuters), and CINAHL Plus with full text (Ebsco) databases. All searches were limited to English-language studies published from 1989 onward with no restrictions on geographical location. Each search was conducted between November 2023 and May 2024, aligned with the respective PICO question. Reference lists of included studies were reviewed to identify further relevant citations.

Public comments & supplemental search

The public comment period for the NBS guideline was open from September 17, 2024 to Oct 27, 2024. Emails were sent directly to CF pediatric program directors and program coordinators, and to genetic counselors at CF programs. A direct email was also sent to a list of state newborn screening lab contacts, and committee members sent the announcement to national discipline-specific groups, nonprofit organizations, and external CF organizations. Reminder emails were sent before the closing of the public comment period. The public comment draft was also presented in sessions at the North American CF Conference and was presented as a poster at the Association of Public Health Laboratories Newborn Screening Symposium, including a rapid-fire poster presentation.

Simultaneously, a supplemental literature search was conducted in PubMed to capture any newly published literature since the initial searches were performed. The articles returned can be found in the online supplement. The reviewers for each PICO question reviewed this additional literature and highlighted any articles that should be included in the final manuscript. There was a total of 84 respondents during the public comment period, in addition to two letters sent directly to the authors. Respondents were asked to select their role from a prepopulated list and check all that apply. Of these respondents, 16 identified as a person living with CF (PwCF), a Caregiver of a PwCF or a Family Member of a PwCF.19 identified as NBS or Public Health Lab Staff, and 74

identified as clinical care providers. 11 respondents selected "other." The manuscript was revised based on public comment.

The CFF intends for these consensus recommendations to provide evidence-based guidance to clinicians, patients, NBS programs, pediatricians, and other stakeholders. These consensus recommendations are intended to be location-agnostic and are applicable to newborns screened for CF throughout the US. In contrast to other CFF guidelines, this guideline has recommendations pertinent to public health practice.

Data availability statement

Full details of the systematic reviews, including spreadsheets for each PICO question with a list of reviewed manuscripts, review metrics, and inclusion/exclusion status, are available upon request from the corresponding author. Please contact Meghan McGarry at meghan.mcgarry@seattlechildrens.org.

PICO 1

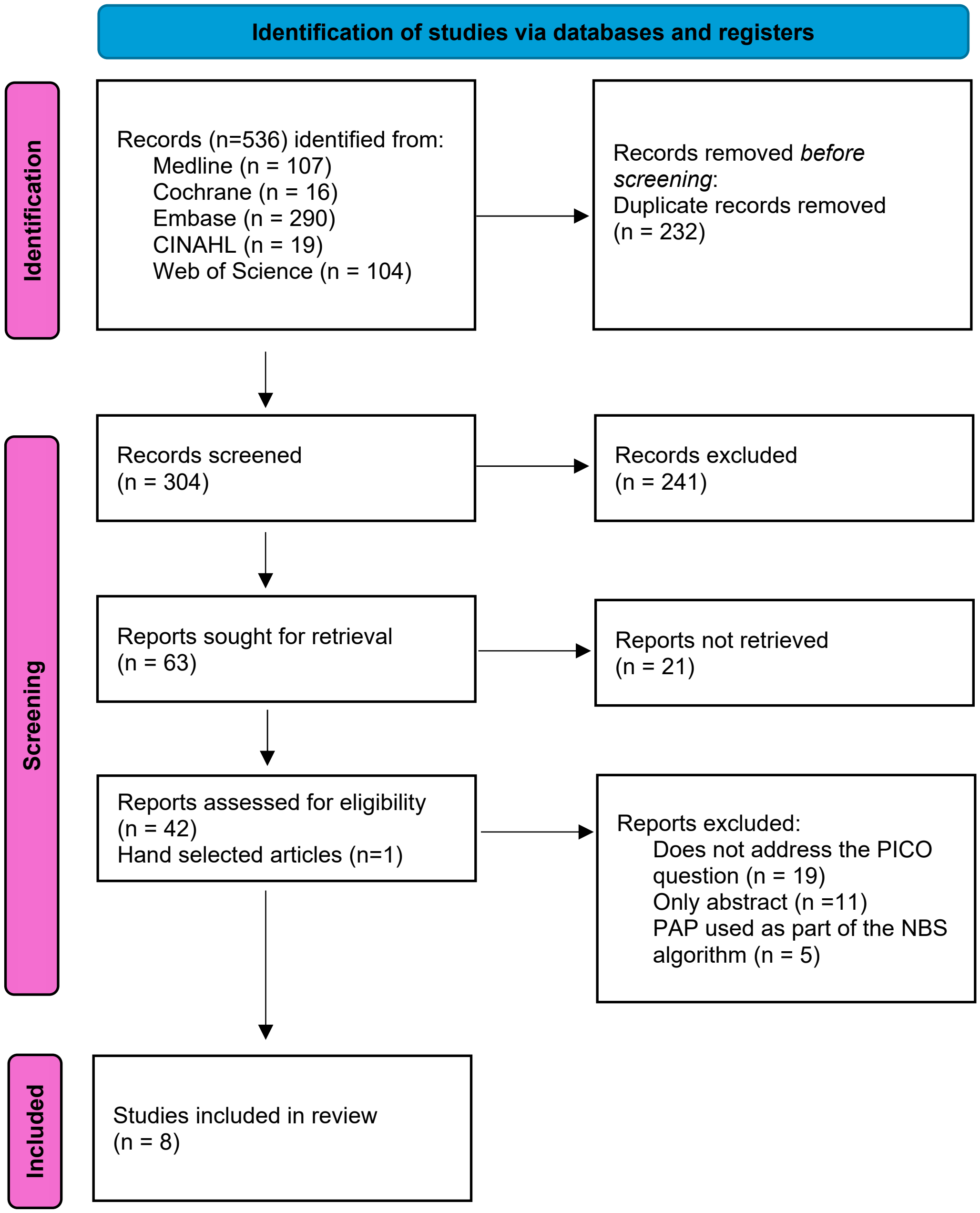

From: Page MJ, McKenzie JE, Bossuyt PM, Boutron I, Hoffmann TC, Mulrow CD, et al. The PRISMA 2020 statement: an updated guideline for reporting systematic reviews. BMJ 2021;372:n71. doi: 10.1136/bmj.n71

For more information, visit: http://www.prisma-statement.org/

PICO 2

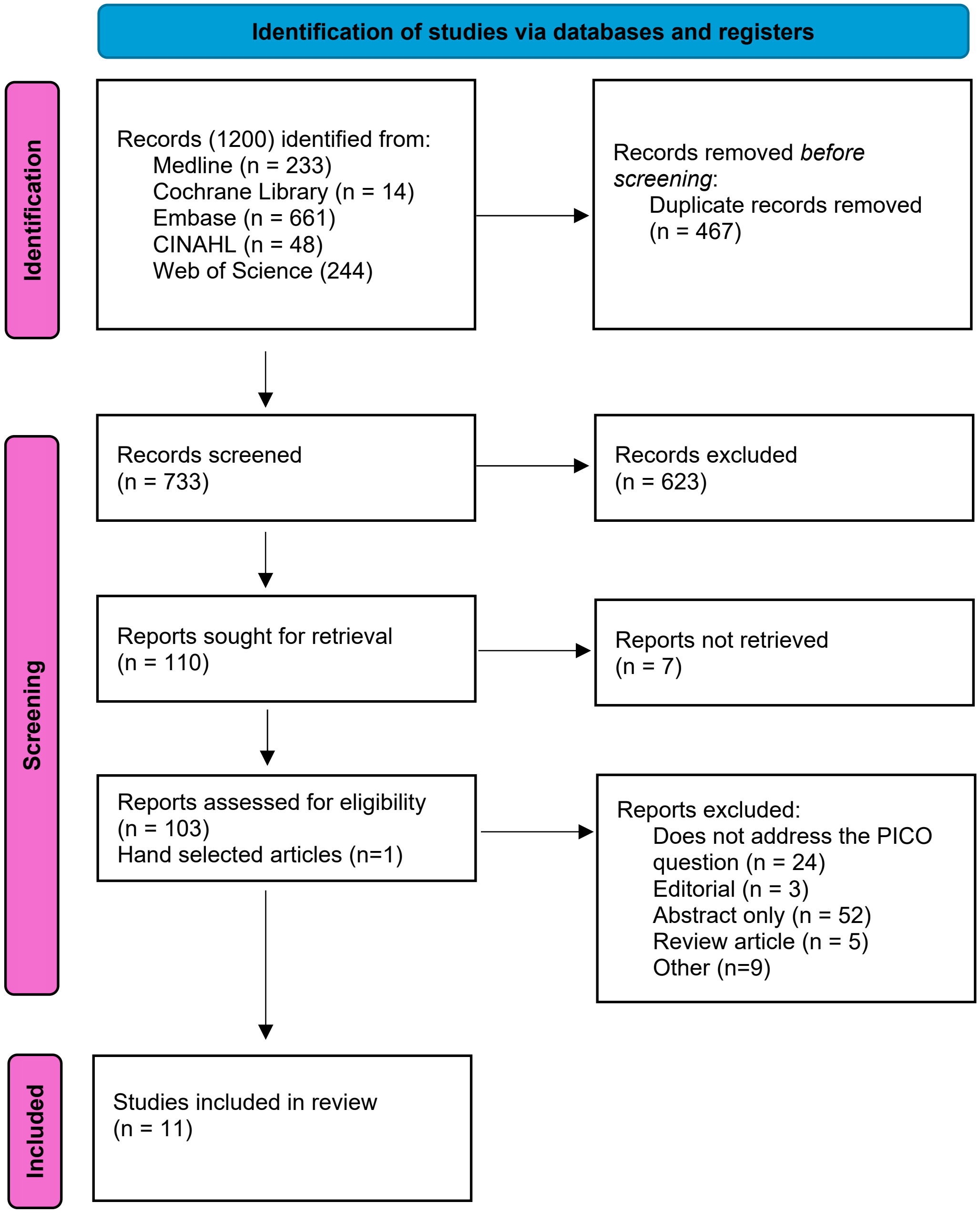

From: Page MJ, McKenzie JE, Bossuyt PM, Boutron I, Hoffmann TC, Mulrow CD, et al. The PRISMA 2020 statement: an updated guideline for reporting systematic reviews. BMJ 2021;372:n71. doi: 10.1136/bmj.n71

For more information, visit: http://www.prisma-statement.org/

PICO 3

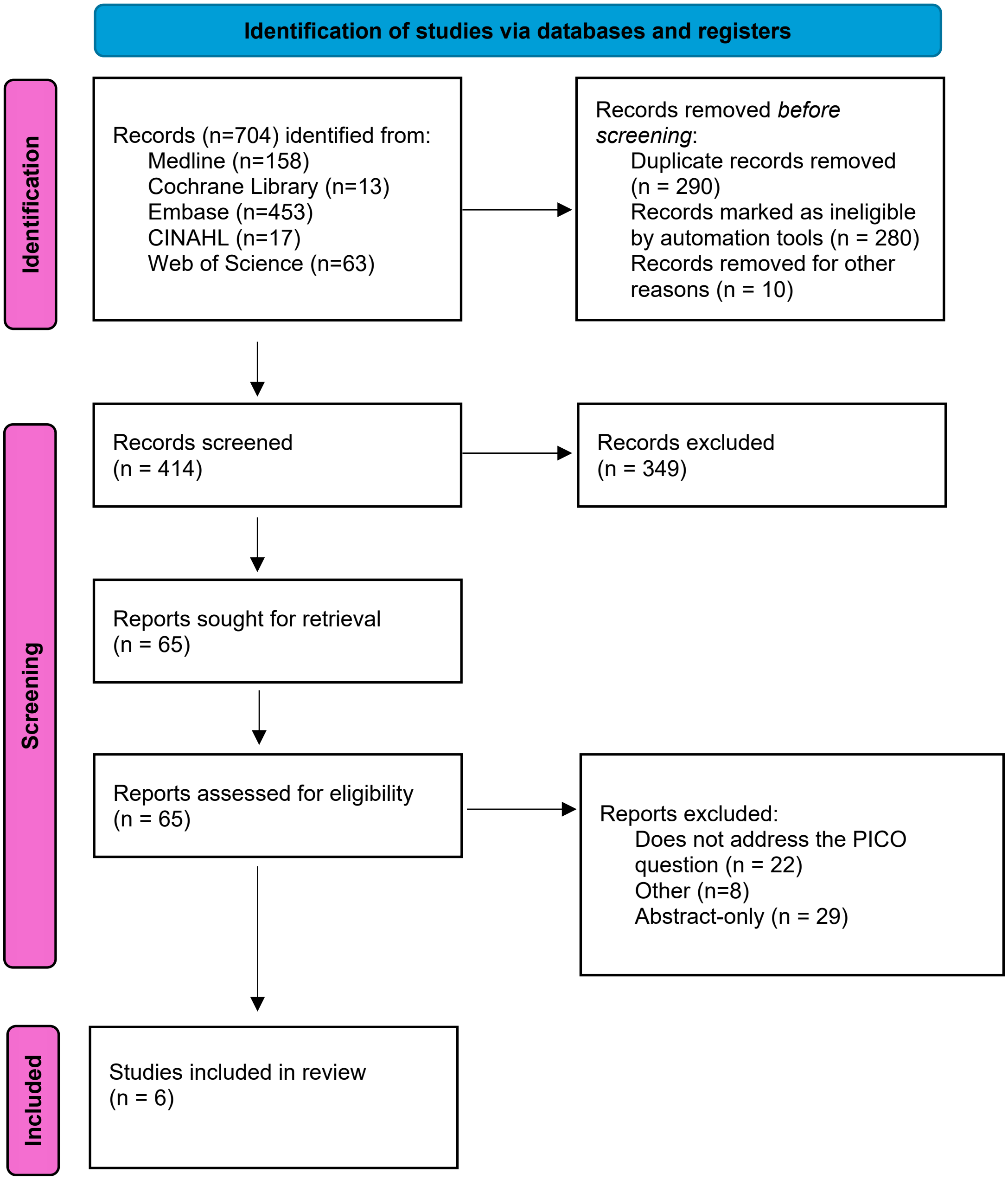

From: Page MJ, McKenzie JE, Bossuyt PM, Boutron I, Hoffmann TC, Mulrow CD, et al. The PRISMA 2020 statement: an updated guideline for reporting systematic reviews. BMJ 2021;372:n71. doi: 10.1136/bmj.n71

For more information, visit: http://www.prisma-statement.org/

PICO 4

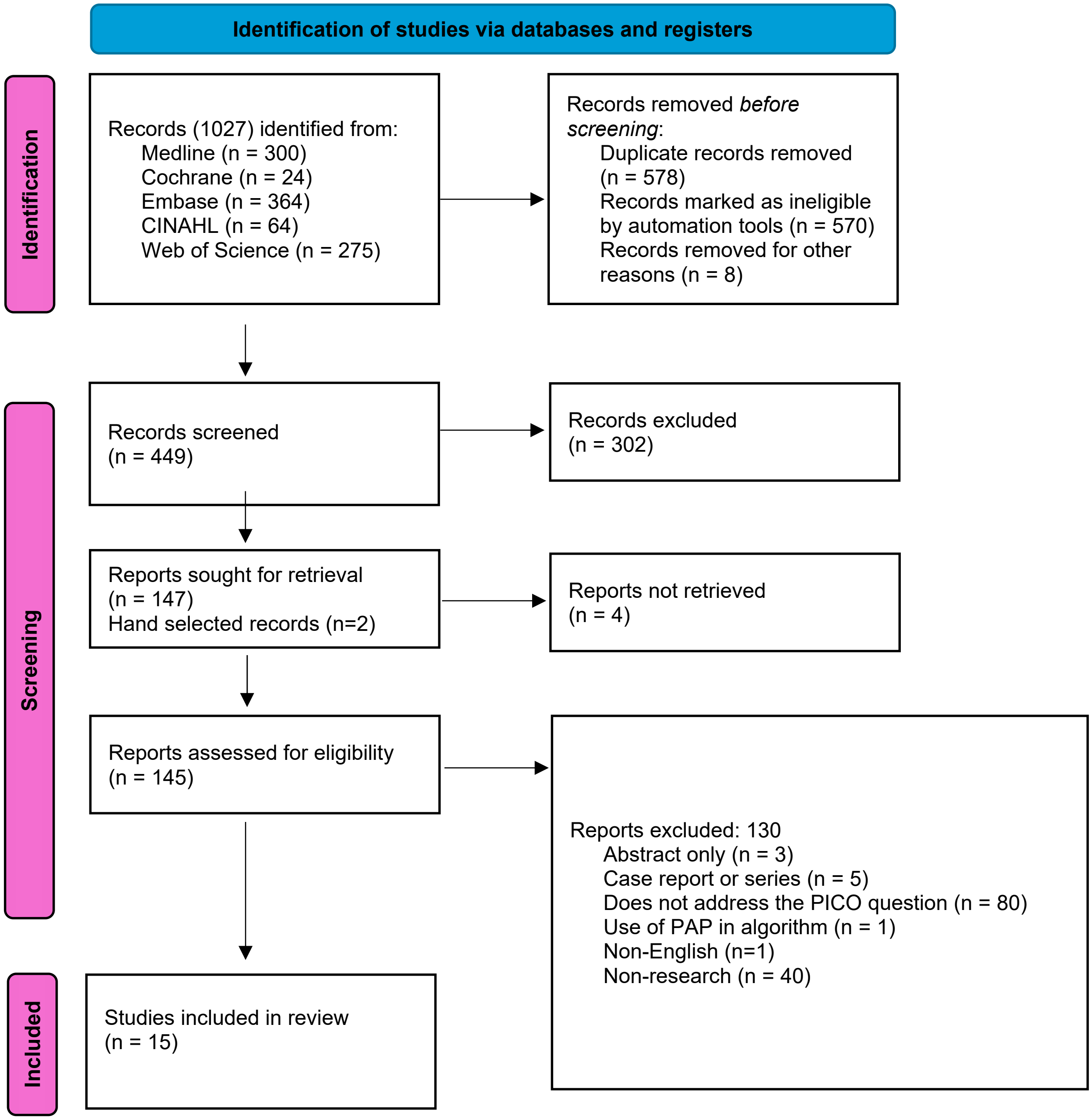

From: Page MJ, McKenzie JE, Bossuyt PM, Boutron I, Hoffmann TC, Mulrow CD, et al. The PRISMA 2020 statement: an updated guideline for reporting systematic reviews. BMJ 2021;372:n71. doi: 10.1136/bmj.n71

For more information, visit: http://www.prisma-statement.org/

PICO 5

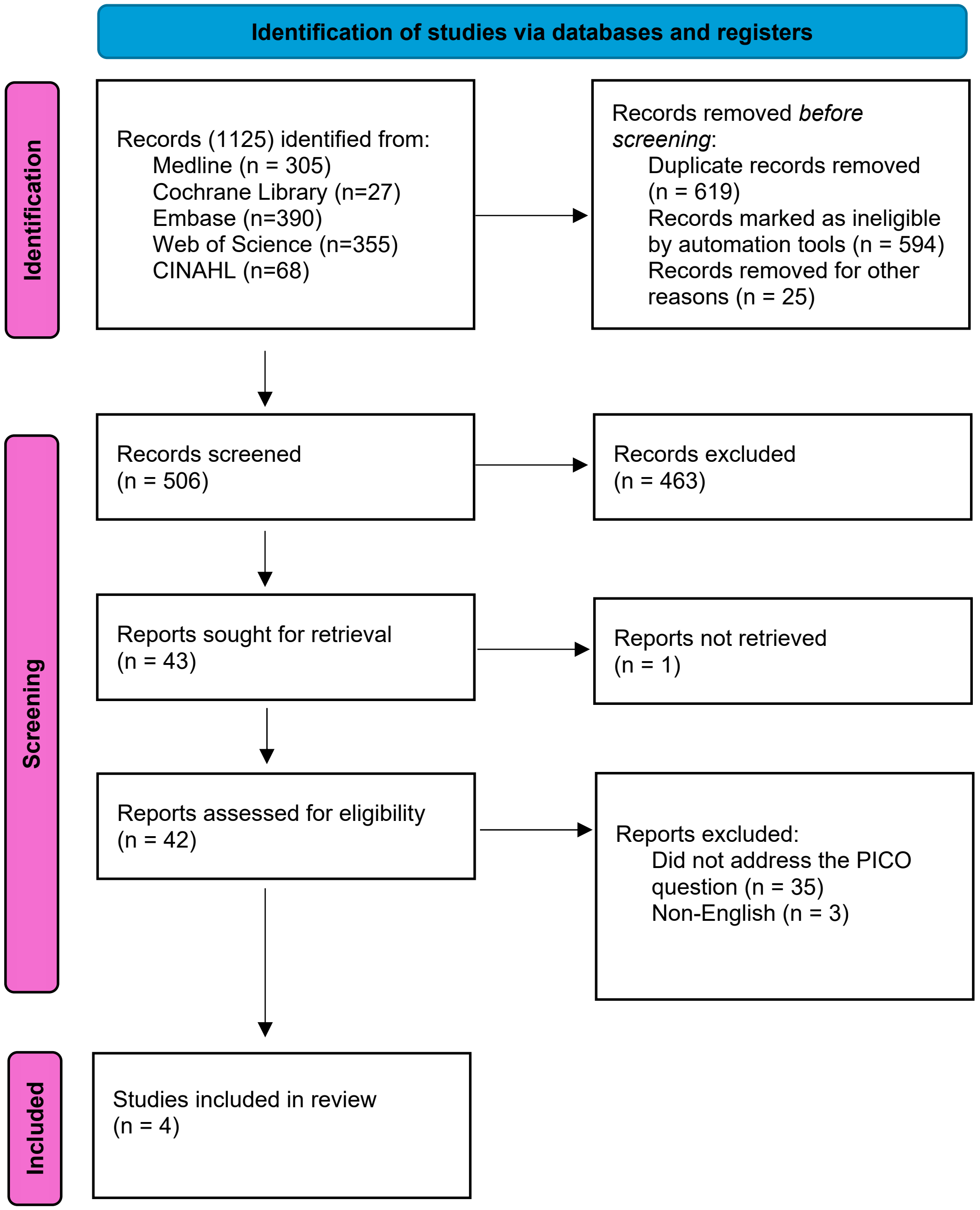

From: Page MJ, McKenzie JE, Bossuyt PM, Boutron I, Hoffmann TC, Mulrow CD, et al. The PRISMA 2020 statement: an updated guideline for reporting systematic reviews. BMJ 2021;372:n71. doi: 10.1136/bmj.n71

For more information, visit: http://www.prisma-statement.org/

PICO 6

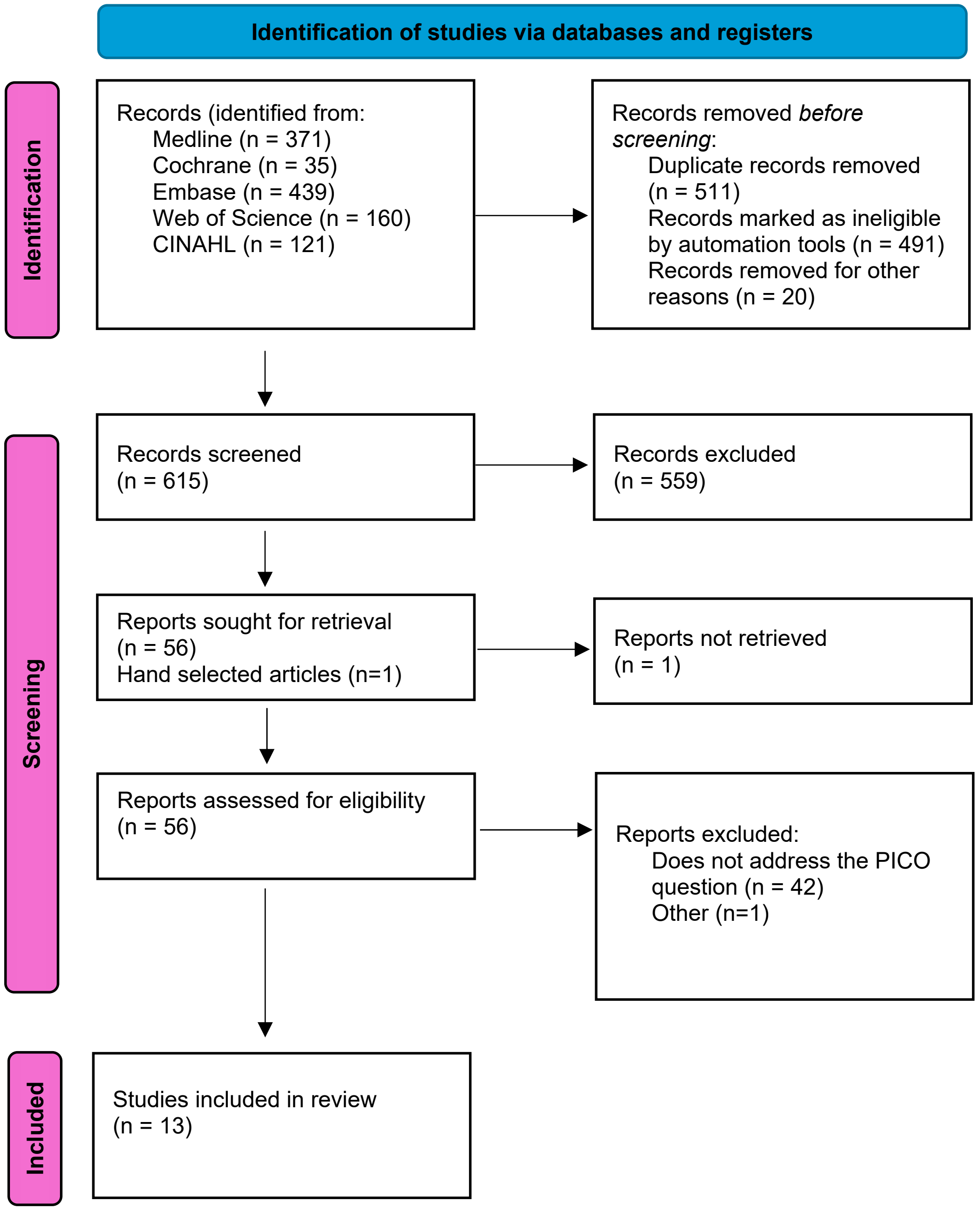

From: Page MJ, McKenzie JE, Bossuyt PM, Boutron I, Hoffmann TC, Mulrow CD, et al. The PRISMA 2020 statement: an updated guideline for reporting systematic reviews. BMJ 2021;372:n71. doi: 10.1136/bmj.n71

For more information, visit: http://www.prisma-statement.org/

References

1. Susanna McColley, Marci Sontag, Karen Raraigh, Meghan McGarry, Marissa Rollins, Q. Eileen Wafford. Very-high IRT utilization in the cystic fibrosis newborn screening algorithm. PROSPERO 2023 CRD42023481926
   Available from:
   https://www.crd.york.ac.uk/prospero/display_record.php?ID=CRD42023481926
2. Susanna McColley, Marci Sontag, Karen Raraigh, Meghan McGarry, Marissa Rollins, Q. Eileen Wafford. Changes in sensitivity of CF newborn screening algorithms when CFTR sequencing follows IRT and CFTR variant testing. PROSPERO 2024 CRD42024500201
   Available from:
   https://www.crd.york.ac.uk/prospero/display_record.php?ID=CRD42024500201
3. Susanna McColley, Marci Sontag, Clement Ren, Christine Dorley, Philip Farrell. Sensitivity of Floating Immunoreactive Trypsinogen Cutoffs in the Cystic Fibrosis Newborn Screening Algorithm. PROSPERO 2024 CRD42024522404
   Available from:
   https://www.crd.york.ac.uk/prospero/display_record.php?ID=CRD42024522404
4. Marissa Rollins, Susanna McColley, Marci Sontag, Karen Raraigh, Meghan McGarry, Q. Eileen Wafford. Sensitivity of the cystic fibrosis newborn screening algorithm among newborn screening programs using expanded DNA variant panels. PROSPERO 2024 CRD42024535075
   Available from:
   https://www.crd.york.ac.uk/prospero/display_record.php?ID=CRD42024535075

5. Marissa Rollins, Susanna McColley, Marci Sontag, Karen Raraigh, Meghan McGarry, Q. Eileen Wafford, Debra Freedenberg, M. Christine Dorley, Cambrey White, Steven Hicks, Philip Farrell, Clement Ren, Karey Padding, Kathryn Tullis. Timeliness of age of first cystic fibrosis encounter among CF newborn screening programs that use algorithms with CFTR DNA testing at least twice a week. PROSPERO 2024 CRD42024542279
   Available from:
   https://www.crd.york.ac.uk/prospero/display_record.php?ID=CRD42024542279
6. Marissa Rollins, Susanna McColley, Marci Sontag, Karen Raraigh, Meghan McGarry, Q. Eileen Wafford, Debra Freedenberg, M. Christine Dorley, Cambrey White, Steven Hicks, Philip Farrell, Clement Ren, Karey Padding, Kathryn Tullis. Influences of newborn screening program notification practices on age of first cystic fibrosis encounter. PROSPERO 2024 CRD42024552545
   Available from:
   https://www.crd.york.ac.uk/prospero/display_record.php?ID=CRD42024552545
7. Green DM, Lahiri T, Raraigh KS, et al. Cystic Fibrosis Foundation Evidence-Based Guideline for the Management of CRMS/CFSPID. *Pediatrics*. 2024;153(5):e2023064657. doi:10.1542/peds.2023-064657
